# Formation of 6-, 8- and 10-Shogaol in Ginger through Application of Different Drying Methods: Altered Antioxidant and Antimicrobial Activity

**DOI:** 10.3390/molecules23071646

**Published:** 2018-07-05

**Authors:** Ali Ghasemzadeh, Hawa Z.E. Jaafar, Ali Baghdadi, Amin Tayebi-Meigooni

**Affiliations:** 1 Department of Crop Science, Faculty of Agriculture, Universiti Putra Malaysia, Serdang 43400, Selangor, Malaysia; hawazej@upm.edu.my (H.Z.E.J.); ali_baghdadi@upm.edu.my (A.B.); 2 Department of Food Science and Technology, Tehran North Branch, Islamic Azad University, Tehran 1987973133, Iran; amin_ir@hotmail.com

**Keywords:** ginger, shogaol, gingerol, hot air drying, antioxidant activity, antimicrobial activity

## Abstract

Gingerols and shogaols are compounds found in ginger (*Zingiber officinale* Roscoe); shogaols are found in lower concentration than gingerols but exhibit higher biological activities. This work studied the effects of different drying methods including open sun drying (OSD) solar tunnel drying (STD) and hot air drying (HAD) with various temperature on the formation of six main active compounds in ginger rhizomes, namely 6-, 8-, and 10-gingerols and 6-, 8-, and 10-shogaols, as well as essential oil content. Antioxidant and antimicrobial activity of dried ginger was also evaluated. High performance liquid chromatography (HPLC) analysis showed that after HAD with variable temperature (120, 150 and 180 °C), contents of 6-, 8-, and 10-gingerols decreased, while contents of 6-, 8-, and 10-shogaol increased. High formation of 6-, 8-, and 10-shogaol contents were observed in HAD (at 150 °C for 6 h) followed by STD and OSD, respectively. OSD exhibited high content of essential oil followed by STD and HAD method. Ginger-treated with HAD exhibited the highest DPPH (IC_50_ of 57.8 mg/g DW) and FRAP (493.8 µM of Fe(II)/g DM) activity, compared to STD and OSD method. HAD ginger exhibited potent antimicrobial activity with lower minimum inhibition concentration (MIC) value against bacteria strains followed by STD and OSD, respectively. Ginger extracts showed more potent antimicrobial activity against Gram positive bacteria than Gram negative bacteria strains. Result of this study confirmed that conversion of gingerols to shogaols was significantly affected by different drying temperature and time. HAD at 150 °C for 6 h, provides a method for enhancing shogaols content in ginger rhizomes with improving antioxidant and antimicrobial activities.

## 1. Introduction

Ginger (*Zingiber officinale* Roscoe) is a well-known spice belonging to the family Zingiberaceae, and is cultivated across the world but especially in Asian countries [1]. Several phytochemicals are found in ginger, including 4-, 6-, 8-, 10-, and 12-gingerols and 6-, 8-, and 10-shogaols, flavonoids, and phenolics [2]. Previous studies have revealed that shogaols are the dehydration products of gingerols and exhibit higher biological activities including anticancer and antioxidant activities [3,4]. Gingerols are the principal constituents of fresh ginger roots, whereas shogaols are found in low amounts in dried and thermally treated roots [5,6]. Previous study by current authors on other ginger variety (var. *rubrum* Theilade) showed that vacuum oven drying at 45 °C resulted in increasing of 6- and 8-shogaol content and antioxidant activity compared to shade (average temperature of 15 °C) and freeze (−60 °C) drying method [7]. Different methods and techniques such as microwave drying, freeze drying, infrared drying, solar drying, oven drying, and fluidized bed dryer are developed for dehydration of ginger [7,8,9]. Some of these drying methods need to specific equipment and are costly. Artificial dehydration methods are used at the industrial scale, but significantly affect the phytochemical composition and biological activity of the plants because of enzymatic and/or non-enzymatic processes that occur during drying [10,11]. Dehydration by sun is cheap, and then sun drying is the popular method for farmer. The major drawbacks of sun drying (conventional drying method) are dependence on the weather, long drying time, microbial contamination, and low quality of the final products [12]. Then, to avoid the weather dependence and reduce the treatment time, and microbial contamination other drying methods should be introduce in large scale and industry to produce high quality dry rhizomes. Compared with traditional drying methods such as solar drying, hot air drying, and steaming, novel thermal technologies have been studied in attempts to achieve faster drying and improve the quality of fruits and vegetables [13,14]. However, hot air-heated pumpkin slices showed a higher rehydration capacity than those dried by other methods (e.g., microwave) [15,16]. Therefore, proper selection of the drying method is necessary to optimize quality of the finished product. To the best of our knowledge, there is dearth of information on the conversion of gingerols to shogaols in ginger during drying process and its effect on antioxidant and antimicrobial activity. This study assessed effect of different conventional drying methods including open sun drying (OSD) solar tunnel drying (STD) and hot air drying (HAD) and their effects on ginger rhizome quality in terms of 6-, 8-, 10, and 12-gingerol and 6-, 8-, 10, and 12-shogaol contents, essential oil content and antioxidant and antimicrobial activities.

## 2. Results and Discussion

### 2.1. Comparison of Gingerols and Shogaols Content between OSD, STD and HAD Method at Different Temperature

The contents of gingerols and shogaols in different drying method and temperature are shown in Table 1. OSD ginger exhibited higher contents of 6-, 8-, and 10-gingerols than STD and HAD-treated ginger (*p* < 0.05). Meanwhile, STD/HAD-treated ginger exhibited considerably higher levels of 6-, 8-, and 10-shogaols than OSD ginger (*p* < 0.05). HAD treatment, decreased gingerol contents in ginger significantly, but induced 6-, 8- and 10-shogaol formation. Result of this study clearly showed that the conversion of gingerols to shogaols in ginger greatly depended on temperature rate. In HAD method with increasing of temperature rate from 120 °C to 150 °C, concentration of 6- and 8-gingerols increased significantly, but interestingly, 10-gingerol, which was identified at 120 °C, was not detected in 150 °C and 180 °C. With increasing of temperature from 120 °C to 150 °C concentration of 6-, 8- and 10-shogaols also dramatically enhanced. Future increasing in drying temperature to 180 °C resulted in decreasing of all gingerols and shogaols content. These results confirm the impact and advantage of temperature in the conversion of gingerols to shogaols, which is consistent with the results of Huang et al. [17] and Cheng et al. [18]. The results indicate that conventional sun dried ginger containing highest content of gingerols and lowest content of shogaols compared to HAD method. HAD involves the exposure of a product to a continuous air flow to remove moisture. This process is complex because of the different mechanisms behind the heat, mass, water, and energy transport processes. The quality of hot air-dried products is often drastically reduced in comparison to the fresh product. HAD provides improved efficiency for the conversion of heat, increased surface drying uniformity, and decreased drying time, when compared to OSD [18]. As opposed to conventional drying methods, HAD directly interacts with the samples. The heat generated in the wet sample due to the direct transmission and absorption of energy by water molecules leads to rapid internal volumetric drying as a result of the friction generated by rotating dipoles and ion movement. In addition, during this process, mass transfer occurs due to the generation of vapor within the product, which is forced to the surface of the product. Bhattarai et al. [3] reported that the conversion rate of gingerols into shogaols depends on temperature rate and duration. This was also proven in this study. Ho and Su [19] studied the effect of heat treatment (75–150 °C) on conversion of gingerol to shogaol in ginger powder after drying process. The authors reported that heating at 150 °C for 80 min, resulted in inducing of 6-shogaol content. However, in previous study oven dried (at 50 °C) ginger powder was treated with different heating temperature, but, in our study we applied different drying method based on different drying temperature in initial step after harvest.

**Figure 1:**
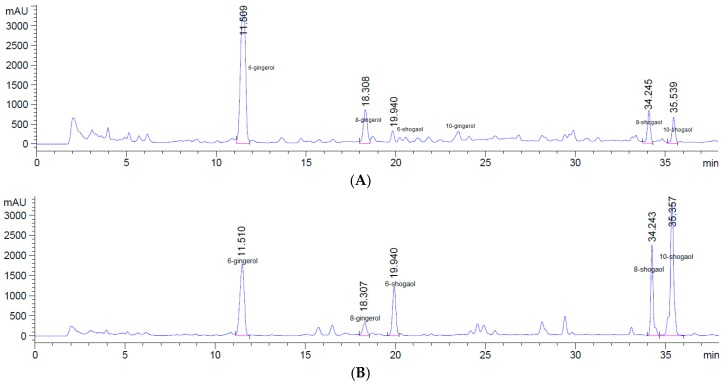
HPLC full chromatogram of ginger extract, dried with OSD (**A**) and HAD (**B**) at 150 °C.

HPLC chromatograms of the extractions of OSD and HAD at 150 °C are shown in Figure 1. As seen, OSD ginger had higher gingerol contents than HAD ginger, but upon application of HAD, gingerol contents decreased while shogaol contents increased. This result supports our previous finding and hypothesis that under high temperature, gingerols will convert to shogaols via dehydration [20].

The most significant factor in thermochemical conversion processes is temperature [21]. When the temperature was further increased (e.g., 180 °C), shogaol contents significantly decreased (*p* < 0.05). This could be due to the degradation and polymerization of shogaols during the long heating process. These results confirmed the effectiveness of heat treatment in increasing shogaol content in ginger. On one hand, temperature facilitates the conversion of gingerols into the corresponding shogaols. The β-hydroxy ketone groups of the gingerol compounds is dehydrated thermally to form the corresponding shogaol derivatives; the resultant α,β-unsaturated ketones in shogaols are thermodynamically stable [22]. Relatedly, acidic solutions facilitate the dehydration of 6-gingerol to produce 6-shogaol, which is similar to the present findings [23]. In aqueous solutions, these acids form different anions which can catalyze the conversion of gingerols to the corresponding shogaols. The increase in shogaol content could be due to the combined effects of the high temperature and the acidic catalyst; these factors induce the β-hydroxy ketone moiety in gingerols to be thermally labile and to undergo dehydration. At 180 °C, shogaol contents sharply decreased (*p* < 0.05). A previous study has shown that the transformation of gingerols into shogaols occurs in aqueous environments [17]. High temperatures lead to faster evaporation of water, thereby inhibiting this transformation [24]. Consequently, degradation and polymerization of shogaols decreased the shogaol contents. Generally, because of HAD at 150 °C represent highest formation of shogaols, this treatment was selected to evaluate effect of different drying time on shogaol contents.

### 2.2. Effect of Different Drying Time on Shogaol Contents during HAD

**Figure 2:**
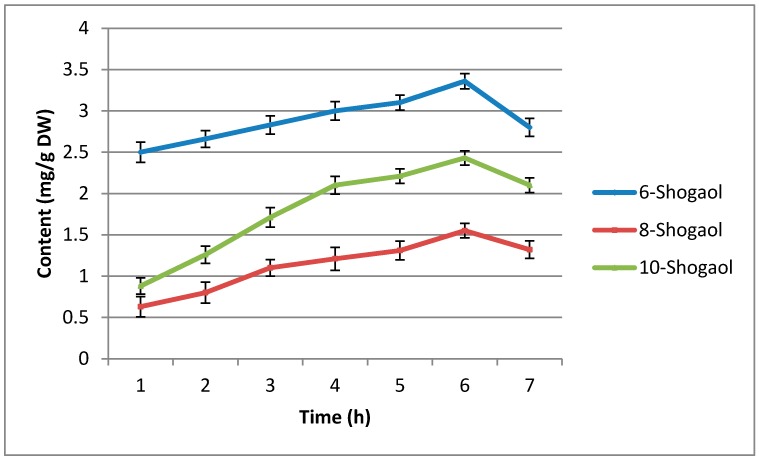
Effects of different drying time on shogaol contents in HAD (at 150 °C) method. Bars indicate standard deviation of triplicate measurements.

In order to evaluation of impact of temperature on shogaol content different drying time was evaluated in HAD (Figure 2). With increasing of drying time from 1 to 6 h, content of shogaols significantly increased, but future increasing of drying time to 7 h, dropt shogaols content significantly. This is due to the degradation and polymerization of shogaols treated for a long time. It has been reported that an increase in temperature leads to a reduction in the water content of SFG [25]. As mentioned previously, the transformation of gingerols into shogaols occurs in an aqueous environment. Consequently, the transformation of gingerols into shogaols was inhibited. Thus, the degradation and polymerization of shogaols caused a decrease in shogaol content [23]. Cheng et al. [18] reported that steam heating treatment (120 °C) for duration of 6 h, induced 6-shogaol content significantly, but after 6 h treatment, content of 6-shogaols slightly decreased.

### 2.3. Effect of Different Drying Method and Temperature on Essential Oil Yield

**Figure 3:**
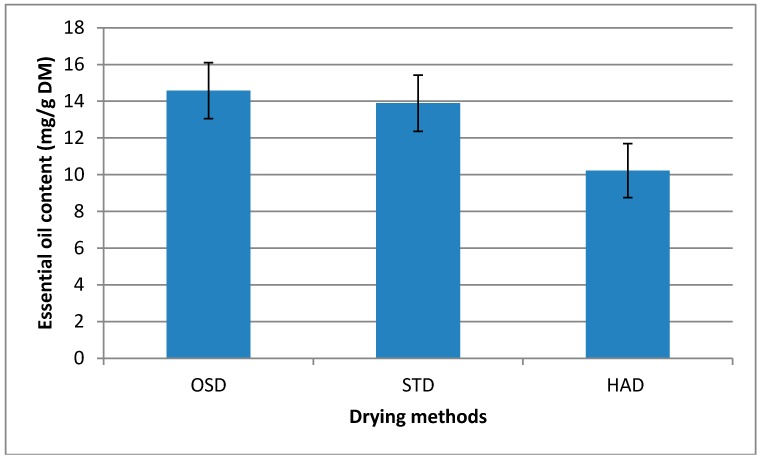
Effect of different drying methods (OSD, STD ant HAD at 150 °C) on essential oil content in ginger. OSD: sun drying, STD: solar tunnel drying and HAD: hot air drying. Bars indicate standard deviation of triplicate measurements.

Content of Essential oil was influenced significantly by different drying methods with variable temperatures. As can see from Figure 3, the highest content of essential oil was obtain from OSD followed by STD and HAD respectively, but, no significant difference between OSD and STD was observed. In other hands with increasing of drying temperature from 34 °C (in OSD) to 150 °C (in HAD) content of essential oil dropped significantly due to decomposition/evaporation of essential oils. Decreasing of essential oil content with increasing of drying temperature was reported by previous studies. Result of recent study, demonstrated that shade dried *Origanum vulgare* L. and *Origanum onites* L. exhibited highest essential oil yield compared to oven dried samples [26]. In another study, increasing of drying temperature from 30 to 50 °C resulted in decreasing of essential oil content from 1.18% to 0.6% in *Lippia citriodora* L. [27]. Similar result also were reported by Ayyobi et al. [28] who find that drying of *Mentha piperita* L. at lower temperature (in shade) resulted in highest essential oil content compared to drying at high temperature (in oven). Then, result of this study showed that, OSD method could be used in order to get highest essential oil yield, compared to STD and HAD (at 150 °C).

### 2.4. Evaluation of Antioxidant Activity

**Figure 4:**
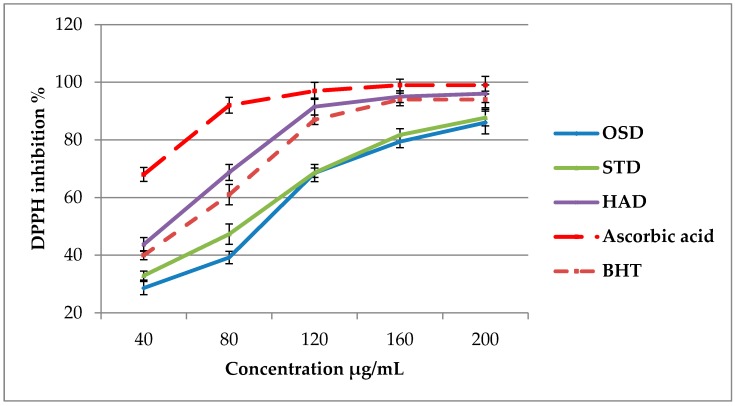
Effect of different drying methods on DPPH activity in ginger extracts. OSD: sun drying, STD: solar tunnel drying and HAD: hot air drying. Bars indicate standard deviation of triplicate measurements.

A substance that offers one or more hydrogen atoms to a radical converting the latter a neutral molecule is referred to as a radical scavenger. This activity is shown as the colour of DPPH turns to yellow from purple (DPPH solution) of the investigated substance because of formation of neutral molecule of DPPH-H upon donation of H atom from the substance. Free radical scavenging ability and reducing power activity are the most reliable antioxidant systems frequently used to elucidate the antioxidant activities of natural products. In our study, the free radical scavenging capacity and the ferric reducing power of the studied extracts were assessed based on DPPH and FRAP tests, respectively. DPPH and FRAP assays are shown to be simple, rapid and highly reproducible. The antioxidant activity of ginger rhizomes dried at different temperatures (OSD, STD, HAD at 150 °C) is shown in Figure 4 and Table 2. The HAD-treated ginger showed higher DPPH-scavenging and FRAP activity than the other drying methods. Therefore, with application of higher temperature in drying process antioxidant activity of ginger was enhanced. Based on the results previously discussed, OSD ginger contained the highest gingerol content, while ginger treated by HAD contained the highest shogaol content. Thus, the highest free radical-scavenging ability of the HAD-treated ginger could be related to high shogaol content. It can be hypothesized that shogaols have higher antioxidant power than gingerols [9]. Recently, Sang et al. [29] reported that 6-, 8-, and 10-shogaols exhibited higher anticancer activity against human lung cancer cells (H1299) than 6-, 8-, and 10 gingerols. Generally, power of free radical scavenging in tested samples for both DPPH and FRAP assay was as follow: HAD > STD > OSD. This results show impact of HAD in order to improving antioxidant activity of ginger rhizomes. HAD ginger exhibited potent antioxidant activity compared to Butylated hydroxytoluene (BHT), while, OSD and STD represent lower free radical scavenging power than BHT. All ginger extracts showed lower antioxidant activity compared to ascorbic acid.

In this study, the IC_50_ based on DPPH assays for HAD, STD and OSD-treated ginger were 57.8, 83.2, and 98.6 mg/g DW, respectively. Lower IC_50_ values signify that the extract is able to scavenge an equivalent amount of free radicals at a lower concentration. Then, HAD method can be recommend as a suitable drying method compared to OSD and STD in order to enhance shogaols content and antioxidant activity for future studies.

### 2.5. Evaluation of Antimicrobial Activity

Ginger was reported as a potent antimicrobial agent against both Gram positive and Gram negative bacteria strains [30]. Drying methods have significant effect on antimicrobial activity of ginger [31]. The results of this study showed that HAD extracts had the potent activities against bacteria strains followed by STD and OSD (Table 3). As can see from the result, ginger extract have more potent antimicrobial activity against Gram positive bacteria than Gram negative bacteria strains. This result is consistent with previous studies on antimicrobial activity of ginger [31]. It had observed that the most sensitive pathogen against ginger extracts was *Staphylococcus aureus* followd by *Bacillus cereus*, *Pseudomonas aeruginosa* and *Escherichia coli* respectively. When comparing antimicrobial activity of OSD with HAD, can find that with increasing of drying temperature antimicrobial activity was increased significantly. Result of phytochemical analysis showed that HAD had lower gingerols and higher shogaols compared to OSD. It can thus be suggested that improvement of antimicrobial activity of ginger which is dried with HAD method could be related to high content of shogaols. Further research should be done to investigate the antimicrobial activity of gingerols and shogaols. Lower and higher MIC value was observed in HAD and OSD respectively (Table 4). Result of recent studies demonstrated that antimicrobial activity of ginger varied when treated with different temperature. Ajayi et al. [31] was reported that ginger rhizomes dried with solar box method exhibited higher antimicrobial activity compared to oven and microwave dried ginger. The results of the antifungal study against the six tested strains, *Candida albicans, Geotrichum candidum, Trichophyton rubrum, Aspergillus flavus, Fusarium oxysporum* and *Scopulariopsis brevicaulis* revealed that HAD-treated ginger exhibited potent antifungal activity except against *T. rubrum* and *S. brevicaulis*. OSD and STD-treated ginger showed antifungal activity only against *C. albicans*. Generally, HAD-treated ginger represent highest antifungal activity followed by STD and OSD respectively.

## 3. Materials and Methods

### 3.1. Reagents

Methanol, acetonitrile (HPLC grade), glacial acetic acid, 6-, 8-, and 10-gingerol, and 6-, 8-, and 10-shogaol were purchased from Sigma-Aldrich (Petaling Jaya, Malaysia). Distilled deionized water and ultra-pure water were used throughout the study.

### 3.2. Sample Preparation and Drying Methods

Fresh ginger rhizomes were collected from herbal farm located in Islamic Azad university, Tehran, Iran. All rhizomes were washed with clean water and were cut into slices. For drying, 500 g of ginger slices were dried using three different methods with different temperature including OSD, STD and HAD methods. For OSD, rhizomes were dried in open yard with direct sun radiation for 9 days (minimum and maximum temperature was 23 ± 1 °C and 34 ± 2 °C respectively). In STD method, ginger slices were dried in UV-stabilized polyethylene tunnel (3 × 3 m) with the hoop structure (minimum and maximum temperature was 31 ± 1 °C and 47 ± 2 °C respectively). In HAD, ginger slices were dried in an electric convection oven at different drying temperatures (120 °C, 150 °C, and 180 °C). The drying time required at each temperature was determined based on a final moisture content of 10 ± 0.2%.

### 3.3. Preparation of Extracts for HPLC Analysis

Briefly, 0.5 g of ginger powder was accurately weighed, suspended in 50 mL of methanol, and sealed. The sample was extracted twice for 30 min each at 40 °C using an ultrasonic bath. Methanol was removed using a rotary evaporator and the residue was kept at −20 °C for future analysis.

### 3.4. HPLC Analysis

A HPLC system (Agilent 1200, Santa Clara, CA, USA) was used to analyze the mixed standards and ginger extracts. Operation parameters were as follows: C_18_ column (250 × 4.6 mm I.D, 5.0 µm particle size); column oven temperature 48 °C; flow rate 1.0 mL/min; injection volume 20 µL; mobile phase A, water, and B, acetonitrile; Gradient mode 0 min 45% B, 8 min 50% B, 17 min 65% B, 32 min 100% B, 38 min 100% B. Crude ginger extract (5 mg) was dissolved in methanol (5 mL) and filtered through a 0.22 μm nylon filter membrane. Standard solutions of gingerols and shogaols were prepared at 1.0 mg/mL. Concentrations of the compounds in each sample were calculated by comparing their peak areas with the corresponding standards.

### 3.5. Essential Oil Content

Essential oil content of dried ginger with different method was analyzed with hydro distillation method using Clevenger apparatus. Briefly, 100 g of dried ginger was mixed with 1 L distilled water in to conical flask and connected to Clevenger apparatus and heated to the boiling point. After 5 h boiling, oil was separated from aqueous layer [32].

### 3.6. Antioxidant Activity

#### 3.6.1. 2,2-Diphenyl-1-picrylhydrazyl (DPPH) Assay

The extracts were examined for their hydrogen donating abilities against the DPPH stable free radical. Sample extracts and ascorbic acid (as a control) were adjusted to 100 µL volume with methanol, mixed with 3 mL of 0.1 mM DPPH in methanol, and vortexed well. Solutions were incubated in the dark for 30 min. The scavenging activity of the extracts was measured via absorbance at 517 nm against a methanol blank and the extract concentration required to inhibit 50% of the DPPH was determined as the IC_50_ [33]. Ascorbic acid and Butylated hydroxytoluene (BHT) were used as positive controls. The following formula was used to calculate the scavenging activity:

% inhibition = [(absorbance control − absorbance sample)/absorbance control] × 100(1)

#### 3.6.2. Ferric Reducing Antioxidant Potential (FRAP) Assay

FRAPP assay was used for evaluation of antioxidant activity. Briefly, 200 µL of extracts were mixed with 2.0 mL of FRAP reagent (pH = 3.6). Mixture was incubated in a water bath at 25 °C for 30 min. The absorbance of the solution (blue colour) was read against acetate buffer (the blank) at 593 nm. A standard curve was prepared using concentrations of 100–1000 mM of FeSO_4_ × 7·H_2_O [34]. The results are expressed in μM of Fe(II)/g DM.

### 3.7. Antimicrobial Activity

#### 3.7.1. Preparation of the Extracts

Dried ginger rhizomes with different methods were dissolved in dimethyl sulphoxide (DMSO) to obtain different concentrations as follow 6.25, 12.5, 25, 50 and 100 µg/mL. Bacterial strains used in this study were: *Escherichia coli* AUMC NO. B-53, *Pseudomonas aeruginosa* AUMC No. B-73 as Gram-negative bacteria and *Staphylococcus Aureus* AUMC NO. B-54, *Bacillus cereus* AUMC NO. B-52 as Grampositive bacteria. While, *Candida albicans* AUMC No. 418, *Geotrichum candidum* AUMC No. 226, *Trichophyton rubrum* AUMC No. 1804, *Aspergillus flavus* AUMC No. 1276, *Scopulariopsis brevicaulis* AUMC No. 729 and *Fusarium oxysporum* AUMC No. 5119 were used for determination of antifungal activity. Bacterial and fungal strains were clinical isolates obtained from the IAU (Islamic Azad University). All bacterial strains were cultivated on nutrient agar medium and incubated at 37 °C for 24 h, while fungal strains were cultivated in potato dextrose agar at 28 °C for 48 h. Cefotaxime as a reference antibacterial at a concentration of 20 μg/mL.

#### 3.7.2. Antibacterial Activity

Agar cup diffusion method was used to reveal inhibition zones caused by the ginger extracts. For evaluation of antibacterial activity, bacterial cultures were adjusted to 0.5 McFarland turbidity standards and inoculated on nutrient agar plates. Circular wells of 7 mm diameter in the inoculated agar were punched carefully using a sterile cork borer under aseptic conditions. For each well various concentration of extract was applied using sterile micropipette. Plates were incubated at 37 °C for 24 h in the incubators. After incubation the diameter of the clear zone of inhibition surrounding the samples was taken as a measure of the inhibitory power of the sample against the particular test organisms [35]. The assay was carried at three times and any zone of inhibition listed as positive results.

#### 3.7.3. Antifungal Activity

One hundred microliters of inoculums (10^6^ CFU/mL; 0.5 McFarland) of the tested saprophytic fungi equally was spread using a sterile glass spreader onto Sabouraud dextrose agar plates. The plates have been kept to dry, and a sterile borer (7 mm in diameter) was used to punch wells in the agar medium. Thereafter, wells were filled with 50 μL of the plant extract at concentration of 100 mg/mL and allowed to diffuse at room temperature for 2 h. The plates were incubated at 25 °C for 72 h. Sterile DMSO used as negative control while clotrimazole (100 μg/mL) was used as positive control [36].

#### 3.7.4. Determination of MIC

The minimum inhibitory concentration (MIC) of the extracts was resolved for each of the test organisms in triplicates. To 0.5 mL of series diluted concentrations (0.78, 1.56, 3.125, 6.25 and 12.5 µg/mL), 2 mL of nutrient broth was added, then tested organism which previously diluted to 0.5 McFarland turbidity standard (for bacterial isolates) was added to the former mixture. A tube containing nutrient broth only was seeded with the test organisms as described above to act as control. Tubes containing bacterial cultures incubated at 37 °C for 24 h. After incubation, the least concentration of the plant extract that does not allow any visible growth or turbidity of the inoculated test organisms in broth culture was taken as the minimum inhibitory concentration in each case.

### 3.8. Data Analysis

Data was analyzed using SAS (Statistical Analysis System, 9.0, SAS Institute, Cary, NC, USA) version 9.2 and Duncan’s multiple range test with significance at the *p* < 0.05 level. The mean and standard deviation (*n* = 3) of each standard and sample were calculated.

## 4. Conclusions

The most obvious finding to emerge from this study is that with application of high temperature in drying process, gingerols were converted to shogaols and following that antioxidant and antimicrobial activities was improved. HAD method simultaneously increased 6-, 8-, and 10-shogaol contents while, OSD method had higher 6-, 8-, and 10-gingerol contents. OSD and STD possessed highest essential oil content than HAD. Furthermore, HAD method can be used to produce high value-added ginger products compared to OSD and STD methods. Data of current study could be useful for future studies in order to optimization of HAD method using response surface methodology in order to get optimum rate of temperature and drying time.

## Figures and Tables

**Table 1:** Quantification of 6-, 8-, and 10-gingerols, as well as 6-, 8-, and 10-shogaols in ginger dried with different temperature.

SD			HAD		
Gingerols	OSD	STD	120 °C	150 °C	180 °C
6-	13.48 ± 2.18 ^a^	10.11 ± 2.17 ^b^	3.43 ± 0.10 ^d^	4.94 ± 0.00 ^c^	2.68 ± 0.10 ^e^
8-	4.08 ± 0.35 ^a^	3.29 ± 0.66 ^b^	0.31 ± 0.03 ^d^	0.43 ± 0.02 ^c^	0.15 ± 0.00 ^e^
10-	5.93 ± 0.73 ^a^	3.96 ± 0.84 ^b^	0.65 ± 0.07 ^c^	ND	ND
Shogaols					
6-	1.54 ± 0.06 ^e^	2.05 ± 0.13 ^d^	3.02 ± 0.78 ^b^	3.93 ± 0.53 ^a^	2.58 ± 0.11 ^c^
8-	0.21 ± 0.00 ^e^	1.55 ± 0.08 ^d^	2.16 ± 0.26 ^b^	2.62 ± 0.21 ^a^	1.73 ± 0.06 ^c^
10-	0.68 ± 0.04 ^e^	1.03 ± 0.03 ^d^	2.04 ± 0.18 ^b^	3.05 ± 0.33 ^a^	1.41 ± 0.02 ^c^

SD: sun drying; HAD: hot air drying. OSD; open sun drying; STD: solar tunnel drying; Data were the mean ± standard deviation of triplicate measurements. Different superscript lower case letters in each row indicated significant difference at *p* < 0.05 (Duncan’s test).

**Table 2:** FRAP activity of ginger extracts, dried with different methods.

Samples	FRAP (µM of Fe(II)/g DM)
OSD	314.5 ± 8.42 ^e^
STD	407.1 ± 10.06 ^d^
HAD	493.8 ± 12.81 ^b^
Ascorbic acid	806.4 ± 20.16 ^a^
BHT	448.2 ± 12.59 ^c^

SD: sun drying; HAD: hot air drying. Data were the mean ± standard deviation of triplicate measurements. Different superscript lower case letters in each row indicated significant difference at *p* < 0.05 (Duncan’s test).

**Table 3:** Inhibition zone of antimicrobial and antifungal activity of ginger, dried with different methods.

Strains	Concentration (µg/mL)	OSD	STD	HAD	Cefotaxime (20 µg/mL)	Clotrimazole (100 µg/mL)
*B. cereus*	100	14	21	24	25	NT
	50	10	15	21		
	25	8	14	18		
	12.5	7	11	13		
	6.25	6	10	10		
*S. aureus*	100	12	23	26	29	NT
	50	10	18	23		
	25	6	15	19		
	12.5	---	11	14		
	6.25	---	9	12		
*E. coli*	100	8	18	18	22	NT
	50	5	15	16		
	25	---	14	14		
	12.5	---	9	11		
	6.25	---	---	8		
*P. aeruginosa*	100	10	20	20	25	NT
	50	8	17	18		
	25	6	15	16		
	12.5	4	13	14		
	6.25	---	12	13		
*C. albicans*	100	12	16	25	NT	37
*G. candidum*	100	---	---	21	NT	42
*T. rubrum*	100	---	---	---	NT	23
*S. brevicaulis*	100	---	---	---	NT	35
*F. oxysporum*	100	---	---	22	NT	37
*A. flavus*	100	10	14	20	NT	36

Unit is mm. NT: not-tested.

**Table 4:** Minimum inhibition concentration (MIC) of ginger, dried with different methods, against bacteria strains.

Strains	OSD	STD	HAD
*B. cereus*	3.125	1.56	1.56
*S. aureus*	6.25	0.78	0.78
*E. coli*	>12.5	3.125	1.56
*P. aeruginosa*	6.25	1.56	1.56

Unit is µg/mL.
